# Bactericidal Effect and Anti-Inflammatory Activity of *Cassia garettiana* Heartwood Extract

**DOI:** 10.1155/2020/1653180

**Published:** 2020-07-13

**Authors:** Sumalee Panthong, Arunporn Itharat, Suchada Naknarin, Pranporn Kuropakornpong, Buncha Ooraikul, Intouch Sakpakdeejaroen

**Affiliations:** ^1^Department of Applied Thai Traditional Medicine, Faculty of Medicine, Thammasat University, Khlong Nueng, Pathumthani 12120, Thailand; ^2^Centre of Excellence in Applied Thai Traditional Medicine Research (CEATMR), Thammasat University, Khlong Nueng, Pathumthani 12120, Thailand; ^3^Faculty of Medicine, Thammasat University, Khlong Nueng, Pathumthani 12120, Thailand; ^4^Bualuang ASEAN Chair Professorship, Thammasat University, Khlong Nueng, Pathumthani 12120, Thailand

## Abstract

Natural products are used as alternative drugs in traditional medicine to treat infection and inflammation and relieve pain. Heartwood of *Cassia garettiana* Craib has been investigated as an ingredient in Thai traditional medicine for anti-HIV protease, but there is no report on its antibacterial and anti-inflammatory activities. The objectives of this study were to investigate the anti-inflammatory and antibacterial activities, time-kill profile, and main active constituents of an ethanolic extract of *C. garettiana* heartwood. The study followed the generally accepted experimental design. All tests were investigated in triplicate. The heartwood of *C. garettiana* was extracted by maceration with 95% EtOH. The antibacterial activity of the extract and its chemical constituents were determined by their MIC values using resazurin as an indicator. Time-kill profile was determined at 0, 2, 4, 6, 8, 10, 12, and 24 hrs and expressed as log CFU/mL. The anti-inflammatory activity of the extract and its chemical components was investigated by their inhibiting effect on IL-6 and TNF-*α* production by ELISA. The ethanolic extract was analyzed for its chemical constituents by HPLC technique. The ethanolic extract showed both dose- and time-dependent bactericidal effects against *Staphylococcus aureus*, methicillin-resistance *Staphylococcus aureus*, *Staphylococcus epidermidis*, *Escherichia coli*, *Pseudomonas aeruginosa*, *Salmonella* Typhi, *Salmonella* Typhimurium, *Klebsiella pneumoniae*, and *Shigella dysenteriae* with MIC values of 312.5, 312.5, 312.5, 1,250, 2,500, 625, 625, 2,500, and 625 *μ*g/mL, respectively. It showed an inhibiting effect on IL-6 production at concentrations of 12.5 to 100 *μ*g/mL. The main active chemical constituent of *C. garettiana* was piceatannol that showed antibacterial activity against all test bacteria except *P. aeruginosa*. *C. garettiana* showed a broad spectrum of antibacterial activity against both Gram-negative and Gram-positive bacteria. Piceatannol and resveratrol from the plant strongly inhibited IL-6 production. Based on these results, we concluded that the ethanolic extract of *C. garettiana* showed both an antibacterial activity and inhibition of IL-6. Piceatannol is the active constituent of the extract and showed anti-inflammatory and antibacterial activities against Gram-negative and Gram-positive bacteria.

## 1. Introduction

Infection is an invasion of pathogens such as viruses, bacteria, fungi, and protozoa in body tissues [1]. The immune system response to these pathogens is to kill them and protect the host from the microorganisms. The response of immune system leads to acute inflammation of the tissue to remove the pathogen and heal cell damage. Unfortunately, acute inflammation leads to the development of chronic inflammation when it cannot be controlled [2]. Normally, antibiotics are used as first-line drugs to treat bacterial infections [3]. However, antibiotic-resistant bacteria are increasing globally, so new antibacterial agents or antibiotics need to be developed to treat these infections, for example, tigecycline, doripenem, and plant extracts [4–6]. *Cassia garettiana* Craib, a herb belonging to the Caesalpiniaceae family, has been used to treat bacterial infections and inflammation and reduce body heat in Thai traditional medicine. Ethanolic extract of *C. garettiana* has been shown to inhibit HIV-1 protease [7]. The heartwood of *C. garettiana* contained piceatannol, chrysophanol, oxyresveratrol, resveratrol, emodin, aloe-emodin, and rhein [8]. Previous studies have shown the antibacterial and anti-inflammatory activities of these compounds [9–11]. However, the antibacterial and anti-inflammatory activities of the ethanolic extract of *C. garettiana* has not yet been explored. Moreover, the contents of the main active constituents have not been investigated. This study was designed to investigate the antibacterial activity, time-kill profile, anti-inflammatory activity, main active constituents of the ethanolic extract of *C. garettiana,* and effect of chemical constituents of the extract on antibacterial and anti-inflammatory activities.

## 2. Materials and Methods

### 2.1. Plant Materials

The heartwood of *C. garettiana* was collected by a Thai traditional doctor from Samutsakorn Province, Thailand, in February 2019. It was identified by comparison with authentic voucher specimens that were kept in the herbarium of Southern Centre of Thai Medicinal Plants, Faculty of Pharmaceutical Sciences, Prince of Songkla University, Songkhla Province, Thailand. The voucher specimen number was SKP034030701.

### 2.2. Chemical and Reagents

Nutrient agar and Mueller Hinton broth were purchased from Difco, USA. Norfloxacin, vancomycin, piceatannol, and resveratrol were purchased from TCI, Japan. Sodium chloride was obtained from Emsure, USA. Resazurin sodium salt, lipopolysaccharide (LPS), and MTT were purchased from Sigma Aldrich, Germany. Acetonitrile, acetic acid, and dimethyl sulfoxide were purchased from RCI Lab Scan, Thailand. Dulbecco's modified Eagle's medium, fetal bovine serum (FBS), and penicillin/streptomycin were purchased from Gibco, USA. IL-6 and TNF-*α* ELISA kits were purchased from ImmunoTools, Germany.

### 2.3. Antibacterial Activity

#### 2.3.1. Microorganisms Testing

A total of nine bacteria species were obtained from The National Institute of Health of Thailand, including three species of Gram-positive bacteria: *Staphylococcus aureus* ATCC25923, methicillin-resistance *Staphylococcus aureus* (MRSA) DMST20651, and *Staphylococcus epidermidis* ATCC12228 and six species of Gram-negative bacteria: *Escherichia coli* ATCC25922, *Pseudomonas aeruginosa* ATCC9027, *Salmonella* Typhi DMST22842, *Salmonella* Typhimurium ATCC13311, *Klebsiella pneumoniae* ATCC70603, and *Shigella dysenteriae* DMST15111.

#### 2.3.2. Preparation of Extract

The heartwood of *C. garettiana* was washed and dried at 50°C for four days, then ground into powder by a grinding machine (DXFILL Model: DXM1000), and macerated with 95% v/v ethanol for three days at room temperature. The mixture was filtered through Whatman no. 1 paper, evaporated, and dried by a lyophilizer (Labconco Model: FreeZone2.5). The dried ethanolic extract was weighed and stored at −20°C until use.

#### 2.3.3. Determinations of Minimal Inhibitory Concentration (MIC) and Minimal Bactericidal Concentration (MBC)

The MIC value of *C. garettiana* ethanolic extract and its chemical constituents, including piceatannol and resveratrol, were determined using the microtiter plate-based antibacterial assay describe by Sarker et al. [12]. Briefly, the extract was dissolved in ethanol at a concentration of 500 mg/mL and diluted with Muller Hinton broth to a maximum concentration of 10 mg/mL followed by twofold serial dilutions to obtain concentrations ranging between 0.078 and 10 mg/mL in Muller Hinton broth, while piceatannol and resveratrol were prepared at 100 mg/mL in ethanol and diluted in serial twofold dilutions (0.0156 to 2 mg/mL). These solutions were transferred to 96-well plates (50 *μ*L/well) in triplicate. Bacteria that had been grown at 37°C for 24 h were transferred into Muller Hinton broth and adjusted to 0.5 McFarland by McFarland densitometer. Bacteria suspension (50 *μ*L/well) was transferred into each well of the 96-well plate and incubated at 37°C for 20 h. The final concentration ranges of *C. garettiana* extract after inoculation were 0.039 to 5 mg/mL, while the final concentration ranges of piceatannol and resveratrol were 0.0078 to 1 mg/mL. The maximum final concentration of ethanol was 1% v/v per well. After the incubation, resazurin sodium salt solution (1 mg/mL) was added into each well (10 *μ*L/well) and incubated continually at 37°C for 3 h. The lowest concentration with no change of resazurin color was recorded as MIC value. Subsequently, all concentrations with no change of resazurin color were transferred onto nutrient agar and incubated at 37°C for 24 h. The lowest concentration with no growth of bacteria was marked as MBC.

#### 2.3.4. Time-Kill Assay

The time-kill assay was performed using a modified method as previously described [13]. The ethanolic extract was prepared at concentrations equal to the MIC, two times the MIC, and four times the MIC. Each concentration was transferred into a culture tube (2 mL/tube). A 0.5 McFarland inoculum was then prepared and added into each tube (2 mL/tube) and incubated at 37°C in a shaker incubator. The samples were collected at 0, 2, 4, 6, 8, 10, 12, and 24 h to determine the colony-forming unit (CFU). All samples were performed in triplicate and recorded as mean ± standard deviation of log CFU/mL.

### 2.4. Anti-Inflammatory Activity

#### 2.4.1. Cell Culture and Viability

RAW264.7 macrophage cells were cultured in Dulbecco's modified Eagle's medium with 10% FBS, 100 *μ*/mL of penicillin, and 100 *μ*g/mL of streptomycin. Cells were subcultured every four days.

#### 2.4.2. Determination of Cell Survival by MTT Assay

RAW264.7 cells were seeded into a 96-well plate with the concentration of 1 × 10^5^ cells/well and incubated at 37°C in 5% CO_2_ for 24 hours. The medium was subsequently removed and replaced with fresh medium containing various concentrations of *C. garettiana* extract or pure compounds, including piceatannol and resveratrol, and 5 ng/mL of lipopolysaccharide (LPS). The plate was incubated at 37°C in 5% CO_2_ for 24 hours. After incubation, supernatant (100 *μ*L/well) was collected to detect IL-6 and TNF-*α* production by ELISA. The survival of the cells was determined by MTT assay. The MTT solution (10 *μ*L/well) was added into each well and incubated at 37°C in 5% CO_2_ for two hours. Supernatants were then removed and replaced with 0.04 M HCl in isopropanol at 100 *μ*L/well. The absorbance of the content was measured at 570 nm and the result was expressed as mean ± SEM of the percentage of survival.

#### 2.4.3. Evaluation of TNF-*α* and IL-6 Production

ELISA kits were used according to the manufacturer's instructions. The supernatant (100 *μ*L/well) was added into the wells that were coated with a specific capture antibody and incubated at room temperature for two hours. The plate was washed five times with wash buffer followed by the addition of the detection antibody (100 *μ*L/well) into each well and incubated for two hours at room temperature. The plate was again washed five times with wash buffer, and then the poly-HRP-streptavidin (100 *μ*L/well) was added into each well and incubated for 30 minutes at room temperature. Finally, the plate was again washed five times with wash buffer, and the TMB substrate solution (100 *μ*L/well) was added into each well and further incubated for 30 minutes in the dark at room temperature. The stop solution was added into each well and the optical density of the content was measured at 450 nm. All experiments were performed in triplicate and the results were recorded as mean ± SEM of TNF-*α* or IL-6 production. Prednisolone was used as a positive control.

### 2.5. Determination of Chemical Constituents in the Ethanolic Extract of *C. garettiana* by HPLC

Piceatannol and resveratrol contents in the ethanolic extract were analyzed using high-performance liquid chromatography (HPLC) system with ultraviolet-visible (UV-vis) detector and automatic injector. Briefly, the ethanolic extract of *C. garettiana* (5 mg/mL) was weighed and dissolved with 1 mL of acetonitrile in a 2 mL vial. The solution was filtered through 0.45 *μ*m filter before injection. The solution (10 *μ*L) was injected into HPLC column. Chemical constituents of the extract were separated by the C18 reverse phase column (Phenomenax Luna 5 *μ* C18 (2) 100 A analytical column 250 × 4.60 mm 5 microns) with a guard column of the same material. The mobile phase gradient elution consisted of 1% acetic acid in water (A) and acetonitrile (B) using four-stage linear gradient: 0–5 min, 90% A, 5–30 min, 60% A, and 30–35 min, 90% A. The elution was performed at a flow rate of 1 mL/min in 35 min and monitored at 286 nm. The piceatannol and resveratrol peaks in the extract were analyzed in the area under the curve and their amounts were determined using the standard curve. However, the piceatannol and resveratrol peak in the ethanolic extract was confirmed by spiking with standard piceatannol or resveratrol. All experiments were performed in triplicate and presented as mean ± standard deviation of mg/g of crude extract.

### 2.6. Statistical Analysis

Statistical analysis was performed using ANOVA and Tukey's multiple comparison tests. Statistical significance was indicated when *p* value <0.05.

## 3. Results and Discussion

### 3.1. Determination of MIC and MBC of *C. garettiana* Extract

The microtiter plate-based antibacterial assay including resazurin as an indicator was performed to investigate the MIC values of the ethanolic extract against nine species of bacteria. Resazurin that was of a blue color was reduced to resorufin (pink) by active bacteria [14]. The *C. garettiana* extract that showed no resazurin color change indicated inhibition of all bacterial growth. The MIC values of the extract are shown in Table 1. The extract showed that it could inhibit all Gram-positive and Gram-negative bacteria, which included *S. aureus*, MRSA, and *S. epidermidis* with the MIC value of 312.5 *μ*g/mL and the MBC/MIC ratios of 2, 2, and 1, respectively. For Gram-negative bacteria, the extract could kill six species of bacteria with MIC and MBC range of 625–2,500 *μ*g/mL. However, Gram-positive bacteria appeared to be more sensitive *to C. garettiana* extract than Gram-negative bacteria.

Antibiotics commonly used to treat and prevent infections are divided into two categories, that is, bacteriostatic and bactericidal [15]. Antibiotic-resistant bacteria are a global problem, especially multidrug-resistant bacteria [16]. New antibiotics and antibacterial agents that are effective against bacterial infections are being developed to replace the antibiotics that have been reported to cause resistance [5]. Plant extracts and their chemical components, including alkaloid, terpenoid, and coumarin, have been reported to have an antibacterial activity [17]. *C. garettiana* is a Thai medicinal plant that has been reported to inhibit HIV-1 protease activity [7]. Seven compounds, which include piceatannol, chrysophanol, oxyresveratrol, resveratrol, emodin, aloe-emodin, and rhein, have been isolated from the plant and showed anti-HIV-1 protease activity [8].

In our study, we tested the antibacterial activity of *C. garettiana* extract and its chemical compounds. The extract showed activity against *S. aureus*, MRSA, *S. epidermidis*, *E. coli*, *P. aeruginosa*, *Salmonella* Typhi, *Salmonella* Typhimurium, *S. dysenteriae,* and *K. pneumoniae* with the MBC/MIC ratio in the range of 1-2. Antibacterial agents are considered bactericidal when they show the MBC/MIC ratio of no more than fourfold [18]. *C. garettiana* extract showed a bactericidal effect against all test bacteria with the MBC/MIC ratio of less than four.

The antibacterial activity of *C. garettiana* is being reported for the first time. However, other *Cassia* species such as *Cassia fistula* and *Cassia alata* have been reported to have antibacterial activity. The ethanolic and methanolic extract of *C. fistula* flowers inhibited *S. aureus*, *E. coli*, *S. epidermidis*, and *K. pneumoniae* in the MIC range of 5–40 mg/mL [19]. The acetone extract of the root of *C. alata* showed a strong inhibition effect against *Bacillus cereus* with MIC value of 40 *μ*g/mL, while the extracts of its leaves and twigs showed moderate antibacterial activity against *B. cereus*, MRSA, and *S. aureus* with the MIC range of 160–320 *μ*g/mL [20]. Similarly, the ethanolic extract of *C. garettiana* showed antibacterial activity against MRSA and *S. aureus* with MIC value of 312.5 *μ*g/mL. One possibility is that *C. garettiana* may have similar chemical constituents to *C. alata*.

### 3.2. Time-Kill Assay of *C. garettiana* Extract against Nine Species of Bacteria

**Figure 1:**
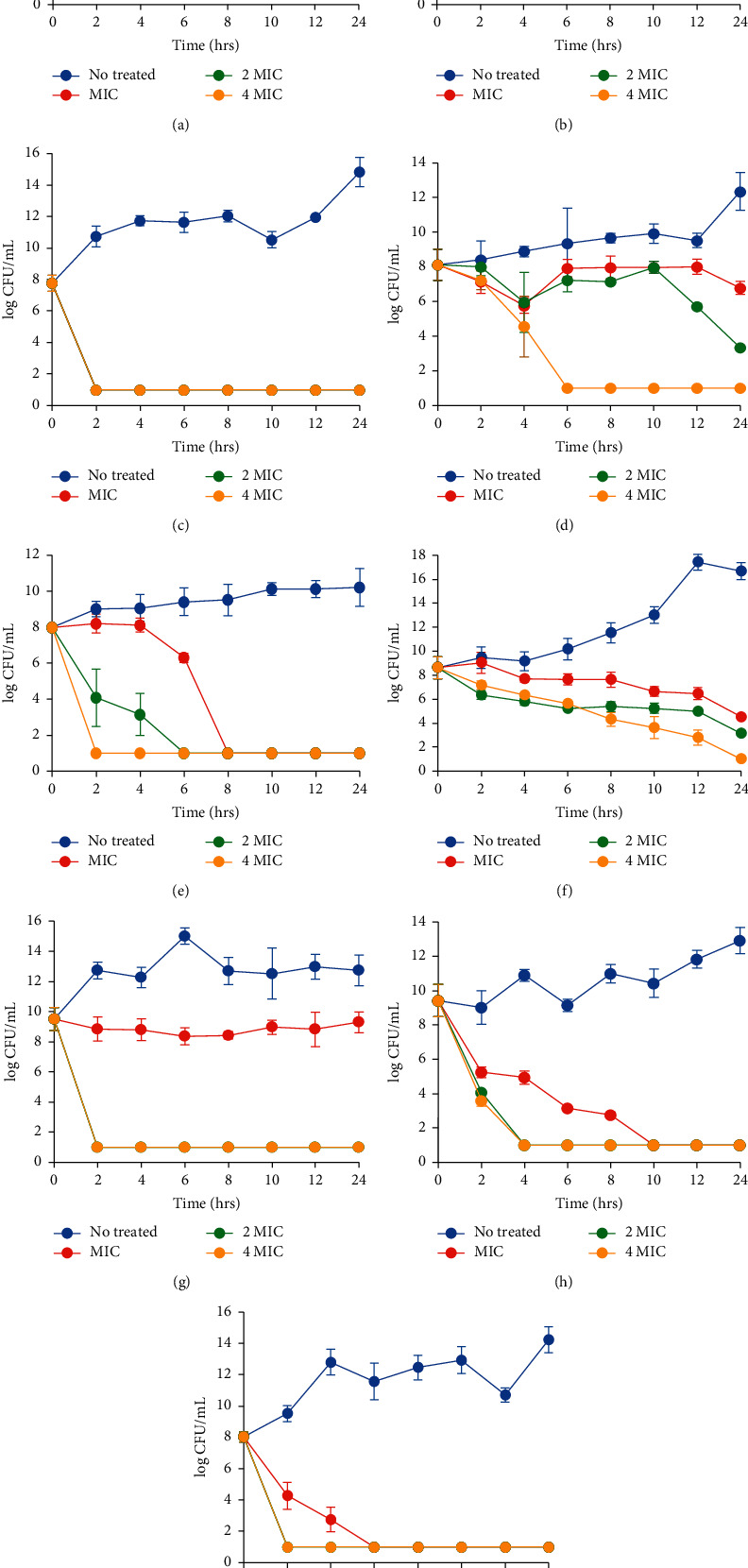
Time-kill plots of *C. garettiana* extract against *S. aureus* (a), *E. coli* (b), *P. aeruginosa* (c), MRSA (d), *S. dysenteriae* (e), *S. epidermidis* (f), *Salmonella* Typhimurium (g), *Salmonella* Typhi (h), and *K. pneumoniae* (i).

The correlation between the concentration and time of antibacterial activity of *C. garettiana* extract is shown in Figure 1. The suppression of the growth of *S. aureus* by the extract at 1X MIC showed a stable time-kill curve. Following treatment for 24 hours, the extract could kill *S. aureus* with a reduction of 2 log10 CFU/mL at 2X MIC and 4X MIC. Similarly, the growth of MRSA and *Salmonella* Typhimurium was inhibited at 1X MIC and killed at 2X and 4X MIC. The ethanolic extract slightly decreased the growth of *S. epidermidis* at 1X MIC and 2X MIC and killed it at 4X MIC, with a reduction of 1 log10 CFU/mL. The reduction of *E. coli*, *S. dysenteriae*, *P. aeruginosa*, *Salmonella* Typhi, and *K. pneumoniae* was 1 log10 CFU/mL when incubated with *C. garettiana* extract at 1X MIC, 2X MIC, and 4X MIC for 2 to 10 h, as shown in Figure 1.

Time-kill assessment is used to confirm the antibiotic's mechanism. Bacteriostatic is defined as the reduction of <3 log10 CFU/mL, while bactericidal is defined as ≥3 log10 reduction in CFU/mL [21]. *C. garettiana* extract showed a dose-dependent bactericidal activity on *S. aureus*, MRSA, *S. epidermidis,* and *Salmonella* Typhimurium. For other bacteria species, the extract showed a time- and dose-dependent bactericidal effect with the reduction of >3 log10 in CFU/mL. The extract showed a broad-spectrum effect against both Gram-negative and Gram-positive bacteria. Broad-spectrum antibiotics are effective against the two bacterial groups [15]. The effect of broad-spectrum antibiotics is not necessarily confined to identified bacteria species, but they also affect nonpathogenic bacteria such as the host-microbiome [22]. *C. garettiana* extract showed an inhibiting effect against MRSA, resistant to *β*-lactam antibiotics that involve the penicillin-binding proteins. Chemical compounds of the plant include epicatechin gallate and epigallocatechin gallate which affect penicillin-binding protein 3 (PBP3) and inhibit the growth of MRSA [23]. Thus, *C. garettiana* extract may affect PBP3 and show an antibacterial mechanism similar to epicatechin gallate in green tea.

### 3.3. Effect of the Ethanolic Extract of *C. garettiana* on TNF-*α* and IL-6 Production in LPS-Stimulated RAW264.7 Cells

**Figure 2:**
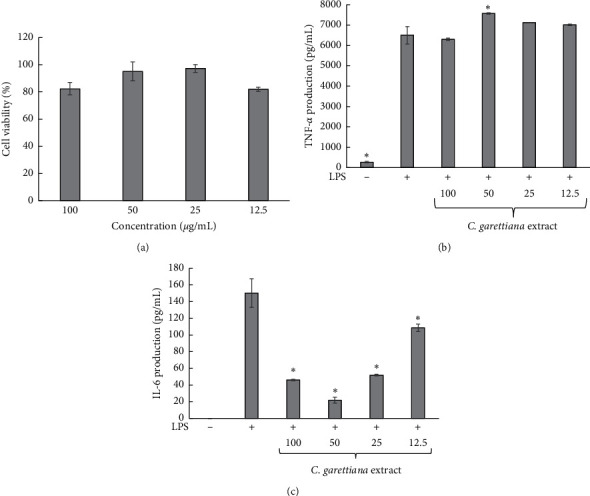
Effect of *C. garettiana* extract on RAW264.7 cell viability (a), TNF-*α* production (b), and IL-6 production (c). ^*∗*^*p* < 0.05 when compared with LPS-stimulated condition.

**Figure 3:**
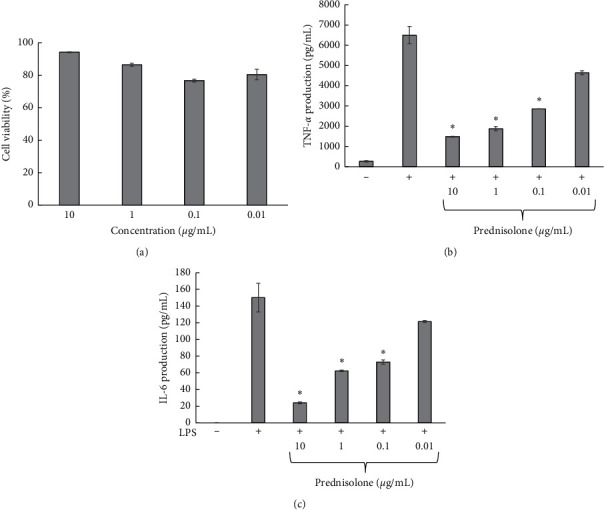
Effect of prednisolone on RAW264.7 cell viability (a), TNF-*α* production (b), and IL-6 production (c). ^*∗*^*p* < 0.05 when compared with LPS-stimulated condition.

The inhibitory effect of the ethanolic extract of *C. garettiana* on proinflammatory cytokine, including TNF-*α* and IL-6, was determined. The results showed no toxicity on RAW264.7 cells where the percentage of cell viability was higher than 70% at all concentrations, as shown in Figure 2(a). *C. garettiana* extract could not inhibit TNF-*α* production, but it significantly inhibited IL-6 production at all concentrations. IL-6 production was lower than 60 pg/mL after treatment with *C. garettiana* extract at the concentrations of 25, 50, and 100 *μ*g/mL, while the extract showed low inhibitory action on IL-6 at the concentration of 12.5 *μ*g/mL. However, prednisolone that was used as a positive control significantly inhibited TNF-*α* and IL-6 production at a concentration of 0.1–10 *μ*g/mL, as shown in Figure 3. TNF-*α* and IL-6 are important cytokines in pathogenic infection. TNF-*α* related host defends it against bacteria and induces fever, while IL-6 induces differentiation of B cells, protects the mucosal, and induces fever [24, 25]. Our work is the first report describing the effect of *C. garettiana* extract on macrophage cytokines. *C. garettiana* extract significantly reduced IL-6 production but did not influence TNF-*α* secretion. IL-6 is known to be a proinflammatory and anti-inflammatory cytokine. The increase of IL-6 expression suppresses inflammation and inhibits IL-1 and TNF-*α* production [26]. The lack of suppression of TNF-*α* production may be related to the decrease of IL-6 production during treatment with *C. garettiana* extract.

### 3.4. Determination of Piceatannol and Resveratrol in the Ethanolic Extract of *C. garettiana* by HPLC

**Figure 4:**
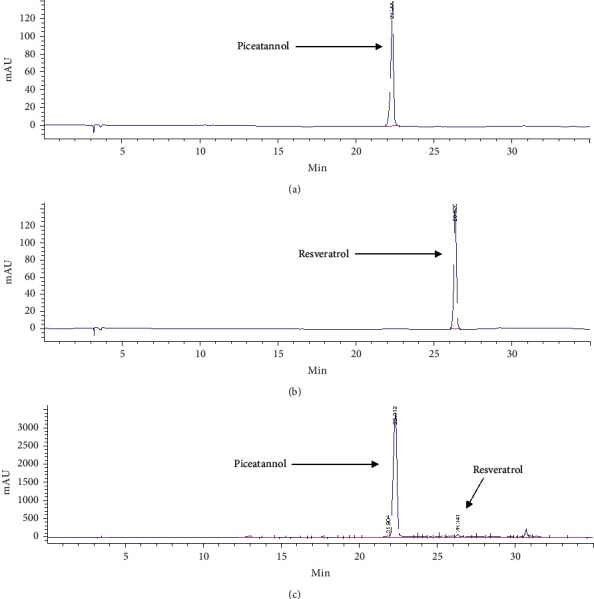
HPLC chromatograms of piceatannol (50 *μ*g/mL) (a), resveratrol (30 *μ*g/mL) (b), and *C. garettiana* extract (5 *μ*g/mL) (c).

**Figure 5:**
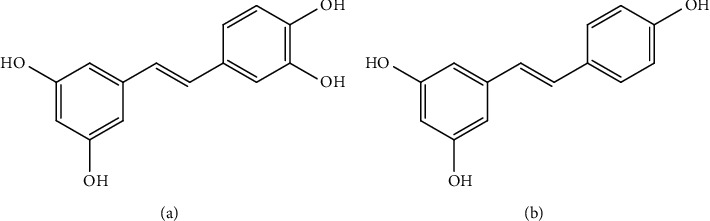
Chemical structures of piceatannol (a) and resveratrol (b).

The ethanolic extract of *C. garettiana* was analyzed for its chemical constituents. The major component found was piceatannol with the retention time of about 22.2 min with the average content of 426.31 ± 17.62 mg/g of dried extract. Resveratrol was another compound found in the ethanolic extract, identified at about 26.3 min with the content of 3.79 ± 0.30 mg/g of dried extract, as shown in Figure 4. The chemical structure of piceatannol and resveratrol is shown in Figure 5.

Piceatannol and resveratrol were investigated for their antibacterial activity. Piceatannol was effective against *S. aureus*, MRSA, and *S. epidermidis* with MIC value of 250 *μ*g/mL, while it showed a less potent activity against *E. coli*, *Salmonella* Typhi, *Salmonella* Typhimurium, *K. pneumoniae,* and *S. dysenteriae* (MIC values: 500–1,000 *μ*g/mL). On the other hand, resveratrol showed no inhibition against any test bacteria, as shown in Table 2.

The major component of *C. garettiana* was piceatannol that showed an antibacterial activity against nine species of bacteria. The extract contained about 42.6% w/w of piceatannol followed by resveratrol. Piceatannol and resveratrol have been reported to possess an antibacterial activity against *Salmonella* Infantis, *Listeria monocytogenes,* and *Candida tropicalis* [27]. Our study also showed that piceatannol could kill two species of *Salmonella*, but resveratrol showed no antibacterial activity. Piceatannol is a hydroxylated analog of resveratrol that contains the hydroxyl group at 3′carbon atom [9]. Some reports showed piceatannol to have better radical scavenging activity than resveratrol because of the hydrogen bond formation of the orthodihydroxy groups [28]. For antibacterial activity, piceatannol showed activity against *P. aeruginosa* while resveratrol can inhibit *S. aureus* with the MIC values of 128 and 512 *μ*g/mL, respectively [29]. Another report showed that piceatannol inhibits *S. aureus* DSMZ6148 with an average MIC of 283 and 383 *μ*g/mL [30]. In our study, piceatannol showed better antibacterial activity than resveratrol. One possibility is that the hydroxyl group may affect bacterial membrane, so piceatannol which has the hydroxyl group at 3′ carbon atom could inhibit bacterial growth better than resveratrol. Resveratrol has been reported to possess antibacterial activity against Gram-negative and Gram-positive bacteria [31]. However, time-kill kinetic of resveratrol displayed bacteriostatic activity towards *S. aureus* [32]. On the other hand, our results showed that resveratrol had no antibacterial activity against all test bacteria. Some reports indicated that a concentration of resveratrol higher than 60 *μ*g/mL reduced the inhibitory effect of resveratrol on growth of mycelia in *Botrytis cinereal* [33]. In this research, resveratrol was prepared at a high concentration that may lead to poor solubility and weak antibacterial activity.

**Figure 6:**
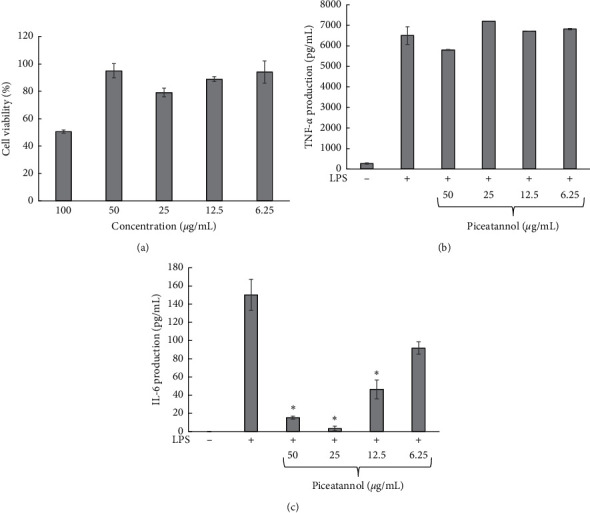
Effect of piceatannol on RAW264.7 cell viability (a), TNF-*α* production (b), and IL-6 production (c); ^*∗*^*p* < 0.05 when compared with LPS-stimulated condition.

In the determination of anti-inflammatory activity, the results showed that piceatannol was toxic on RAW264.7 cells at a concentration of 100 *μ*g/mL, with the cell viability lower than 70%, as shown in Figure 6(a). The inhibitory effect of piceatannol on IL-6 and TNF-*α* production was further investigated at concentrations of 6.25–50 *μ*g/mL. It showed no inhibitory effect on TNF-*α* production, similar to *C. garettiana* extract (Figure 6(b)). However, IL-6 production was lower than 50% of maximum when LPS-stimulated RAW264.7 was treated with piceatannol at the concentrations of 12.5–50 *μ*g/mL. The IL-6 production increased with 6.25 *μ*g/ml of piceatannol when compared with other concentrations (Figure 6(c)).

**Figure 7:**
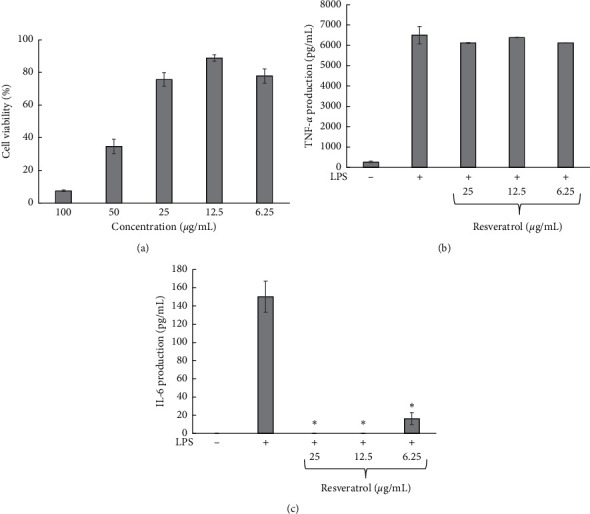
Effect of resveratrol on RAW264.7 cell viability (a), TNF-*α* production (b), and IL-6 production (c); ^*∗*^*p* < 0.05 when compared with LPS-stimulated condition.

Resveratrol was toxic to RAW264.7 cells at the concentrations of 50 and 100 *μ*g/mL; therefore, its inhibitory effect on TNF-*α* and IL-6 was determined at 6.25–25 *μ*g/mL, as shown in Figure 7(a). Results showed that TNF-*α* production was not inhibited by resveratrol at all concentrations (Figure 7(b)), but it completely inhibited IL-6 production from RAW264.7 cells at 12.5 and 25 *μ*g/mL. At 6.25 *μ*g/mL, resveratrol strongly inhibited the IL-6 production but did not completely inhibit its expression, as shown in Figure 7(c).

Piceatannol and resveratrol were chemical constituents found in *C. garettiana*. Piceatannol has been reported to inhibit TNF-*α* and IL-6 production at the concentration of 10–30 *μ*M, while resveratrol could inhibit only TNF-*α* production in LPS-stimulated RAW264.7 cells [34]. Our investigation differs from the previous report in that it showed piceatannol and resveratrol had an inhibition effect on IL-6 but not on TNF-*α*. Other reports showed that piceatannol decreased mRNA expression and protein level of IL-6 and MCP-1in 3T3-L1 adipocytes cells via blocking of I*κ*B*α* phosphorylation and NF-*κ*B activation, while resveratrol also inhibited cytokines and chemokine secretion such as IL-1*α*, IL-1*β*, IL-6, TNF-*α*, CCL4/MIP-1*β,* and CCL5/RANTES in LPS-activated macrophages [35, 36]. On the other hand, our study revealed that resveratrol inhibits only the level of IL-6 production. Similarly, diphlorethohydroxycarmalol that was isolated from a brown alga showed a strong inhibition effect on IL-6 but not TNF-*α* via suppressing Jak2-STAT5 activation and SOCS1 augmentation [37]. Therefore, resveratrol may suppress the specific mechanism in IL-6 production. However, the main active compound of *C. garettiana* was piceatannol that was found to be about 43% w/w of dried extract. Piceatannol and *C. garettiana* extract showed similar antibacterial and anti-inflammatory activities. Other active components in *C. garettiana*, for example, resveratrol, may also affect the biological activity of this plant.

## 4. Conclusions

In conclusion, *C*. *garettiana* was a broad-spectrum bactericidal agent. It contained major bioactive compounds which included piceatannol that showed anti-inflammatory and antibacterial activities against Gram-negative and Gram-positive bacteria. A study on the use of *C. garettiana* extract in combination with antibiotics against serious bacterial infections may prove useful. Furthermore, *C*. *garettiana* showed a high potential for inhibition of MRSA, so its effect on beta-lactamase and other antibiotic-resistant strains of bacteria should be assessed in the future.

## Figures and Tables

**Table 1:** The MIC and MBC values of *C. garettiana* extract, norfloxacin, and vancomycin against nine species of bacteria.

Bacteria	*C. garettiana* extract			Norfloxacin		Vancomycin	
	MIC (*μ*g/mL)	MBC (*μ*g/mL)	MBC/MIC	MIC (*μ*g/mL)	MBC (*μ*g/mL)	MIC (*μ*g/mL)	MBC (*μ*g/mL)
*S. aureus*	312.5	625	2	1.5625	25	NT	NT
MRSA	312.5	625	2	>200	>200	1.5625	12.5
*S. epidermidis*	312.5	312.5	1	0.7813	1.5625	NT	NT
*E. coli*	1.250	1.250	1	0.0122	0.024	NT	NT
*K. pneumoniae*	2.500	2.500	1	1.5625	6.25	NT	NT
*P. aeruginosa*	2.500	2.500	1	0.78	12.5	NT	NT
*S. dysenteriae*	625	625	1	0.024	0.048	NT	NT
*Salmonella* Typhi	625	625	1	1.5625	200	NT	NT
*Salmonella* Typhimurium	625	625	1	0.3906	0.7813	NT	NT

^*∗*^NT = not test.

**Table 2:** MIC and MBC values of piceatannol and resveratrol against nine species of bacteria.

Bacteria	Piceatannol			Resveratrol	
	MIC (*μ*g/mL)	MBC (*μ*g/mL)	MBC/MIC	MIC (*μ*g/mL)	MBC (*μ*g/mL)
*S. aureus*	250	500	2	>1.000	>1.000
MRSA	250	500	2	>1.000	>1.000
*S. epidermidis*	250	250	1	>1.000	>1.000
*E. coli*	500	500	1	>1.000	>1.000
*K. pneumoniae*	1.000	1.000	1	>1.000	>1.000
*P. aeruginosa*	>1.000	>1.000	—	>1.000	>1.000
*S. dysenteriae*	500	500	1	>1.000	>1.000
*Salmonella* Typhi	500	500	1	>1.000	>1.000
*Salmonella* Typhimurium	500	500	1	>1.000	>1.000

## Data Availability

The datasets used and/or analyzed during the current study are available from the corresponding author on reasonable request.
